# Reproductive Performance Following Transcervical Insemination with Frozen Thawed Semen in Ewes Submitted to Surgical Incision of Cervical Folds (SICF): Comparison with Laparoscopic Artificial Insemination

**DOI:** 10.3390/ani10010108

**Published:** 2020-01-09

**Authors:** Salvatore Pau, Laura Falchi, Mauro Ledda, Ivo Pivato, Melosu Valentino, Luisa Bogliolo, Federica Ariu, Maria Teresa Zedda

**Affiliations:** 1 Dipartimento di Medicina Veterinaria, Università degli Studi di Sassari, via Vienna n.2, 07100 Sassari, Italy; nuvola@uniss.it (S.P.); vetleddamauro@gmail.com (M.L.); valentino.melosu@gmail.com (M.V.); luis@uniss.it (L.B.); federica@uniss.it (F.A.); zedda@uniss.it (M.T.Z.); 2 Centro di Competenza Biodiversità Animale, viale Adua 2/c, 07100 Sassari, Italy; 3 Faculdade de Agronomia e Medicina Veterinaria, Universidade de Brasilia, Campus Universitario Darcy Ribeiro, Brasilia 70910-900, Brazil; pivato@unb.br

**Keywords:** surgical incision of cervical folds, lambing, pregnancy, sheep, spermatozoa, transcervical artificial insemination

## Abstract

**Simple Summary:**

The anatomical barriers of the female reproductive tract and the low quality of the frozen thawed semen of rams are limiting factors in spreading superior genotypes in the ovine species. Artificial insemination (AI) is therefore mostly performed by laparoscopy. The surgical incision of cervical folds (SICF) has been demonstrated to allow transcervical intrauterine semen deposition. Here we present data regarding pregnancy (PR) and lambing (LR) rates obtained by transcervical AI following SICF, compared to those obtained by laparoscopic AI using frozen thawed semen. The results showed that in 89.7% of ewes submitted to SICF intrauterine, the deposition of semen was allowed. Moreover, in both experimental groups, the PR and LR were similar. These observations suggest that SICF could represent a valid preliminary procedure to allow transcervical AI in animals of superior genotypes contributing to genetic improvement.

**Abstract:**

Transcervical artificial insemination (AI) after the surgical incision of cervical folds (SICF) could represent a valid alternative to laparoscopic AI when frozen thawed semen is used. The aim of this experiment was to compare pregnancy (PR) and lambing rates (LR) of ewes submitted either to transcervical AI after SICF or to laparoscopic AI using frozen thawed semen. Pregnant at term ewes (n = 80) were allocated in two experimental groups. After lambing, one group (n = 39) was submitted to SICF. The remaining ewes that were regularly lambed were allocated to the group of laparoscopic AI (n = 40). Six months later, oestrous cycle of both experimental groups was synchronised and all ewes were artificially inseminated with frozen thawed semen. Ewes submitted to SICF underwent transcervical insemination and intrauterine deposition of semen was recorded. The remaining animals were submitted to laparoscopic AI. Pregnancy and LR were recorded. Intrauterine deposition of semen was possible in 89.7% pf ewes submitted to SICF. This group showed similar PR and LR compared to the laparoscopic group (respectively: PR, 71.8% vs. 70% and LR, 64.1% vs. 65%; *p* > 0.05). Transcervical AI after SICF may represent a valid alternative to laparoscopy in AI protocols requiring the use of frozen thawed semen.

## 1. Introduction

In the ovine species, artificial insemination (AI) by laparoscopy is the most widely employed technique in breeding programs when frozen thawed (FT) ram semen is used. It allows the deposition of semen directly into the uterine horns, overcoming the two most important limits of AI in the sheep: (i) the convoluted anatomy of the cervix that, differently from other species (bovine, swine, equine), does not allow the passage of an insemination pipette [1]; (ii) the low fertilizing ability of FT spermatozoa, which is easily damaged by oxidative stress due to cold shocks [2]. Pregnancy rates following laparoscopic AI using FT semen are fairly good and comparable to those obtained with fresh semen when cervical insemination is performed [3]. Furthermore, another great advantage is the possibility of using an inseminating dose with lower sperm concentration. Spermatozoa, being deposited closer to the fertilization site, do not face the “obstacles” related to trespassing the cervical barrier. However, it is a surgical procedure requiring anaesthesia, equipment and trained veterinarians.

In the past years, many attempts have been made to overcome the disadvantages of AI in ovine breeding programs, mainly focusing on the cervical barrier. Among others, hormonal treatments inducing cervical relaxation [4,5,6,7,8,9], design of catheters adapted to the tortuous lumen of the cervix [10,11,12,13] and changes in the insemination technique [14] have been attempted in the past decades but the results of these studies are still controversial.

Recently, our research group has presented a new approach for transcervical intrauterine deposition of semen based on surgical incision of the cervical folds (SICF) [15]. This technique represents a novel and encouraging preliminary procedure that is expected will not need to be repeated on the same subject allowing satisfactory pregnancy rates throughout the entire reproductive life of the animal [15]. Moreover, transcervical insemination is easier to perform compared to laparoscopic AI and this characteristic would allow faster spreading of superior genotypes with a positive impact on the number of sheep inseminated worldwide.

Therefore, the aim of the study was to compare the two insemination techniques, laparoscopic AI and transcervical AI following SICF, in terms of pregnancy and lambing rates of ewes inseminated at fixed time with FT semen.

## 2. Materials and Methods

### 2.1. Animal Management

The experiment, carried out in a sheep farm in North Sardinia, started in October 2017 and ended during the breeding season in June 2018. Eighty pregnant, at-term multiparous Sarda ewes (age 3–4 years; body weight ~40 kg) were randomly allocated to one of two experimental groups. One group of ewes (n = 40) was submitted after lambing to SICF. The remaining ewes that were regularly lambed were allocated to the group selected for laparoscopic AI (n = 40). All the experimental procedures were carried out in compliance with European regulations on the Care and Welfare of Animals in Research and were ethically approved by the organism in charge for Animal Welfare and Animal Testing (Organismo Preposto al Benessere Animale ed alla Sperimentazione sugli Animali-OPBSA) of the University of Sassari (protocol number: 26064).

### 2.2. Surgical Incision of Cervical Folds (SICF)

The surgical procedure of incisions of cervical folds has already been described in detail by Pau et al. [15]. Briefly, surgery was performed within 24 h from natural lambing and following confirmation that the fetal membranes had been expelled. Following mild sedation with acepromazine maleate (0.5 mL/50 Kg BW, IM, Prequillan, Fatro S.p.A, Ozzano dell’Emilia, Italy) and epidural anaesthesia with injection of Lidocaine 2% (30 mg/10 kg BW, Esteve S.p.A., Milan, Italy), ewes were placed in dorsal recumbency with the hindquarters slightly elevated. After careful cleansing and disinfection of the perineal area, a lubricated speculum was gently inserted into the vagina and the external *os* of the cervix was localised. The most external fold of the *os* was grasped with Duval forceps and the whole cervix was retracted caudally up to the vaginal *vestibulum*. The remaining folds, up to the most cranial one, were grasped one by one and retracted until complete exteriorization. Afterwards, each fold was incised by electrocautery in four sites: dorsally, ventrally and two laterally. The surgery, performed by an expert veterinary surgeon, lasted around 28 ± 6 min. Local antibiotic treatment was provided and the extruded folds were gently repositioned. Ewes were kept under post-operatory observation for 24 h.

### 2.3. Estrous Synchronisation

Six months after surgery, oestrous cycles of both experimental groups (incision of cervical folds, n = 40; laparoscopic, n = 40) were synchronised by insertion of intravaginal sponges impregnated with fluorogestone acetate (Crono-gest 20 mg, Intervet Italia S.r.l, Segrate, Italy) for 14 days. Before insertion, sponges were dusted with antibiotic powder (sulphanilamide, sulfaguanidine, benzilpenicillin, clortetraciclin; 5–6 g every 25 sponges, Izoaspersorio, Izo, Brescia, Italy). At sponge removal, no abnormal vaginal discharge was observed and ewes received 300IU of eCG (equine chorionic gonadotropin) IM. Teaser rams fitted with marking harnesses were introduced to the experimental flock and 42–48 h after sponge removal, all ewes were marked and hence supposed to be in oestrus. One animal allocated in the SICF group died for causes not related to the experiment.

### 2.4. Artificial Insemination with FT Semen

#### 2.4.1. Semen Preparation

Ejaculates of three fertile rams were collected and assessed for volume, mass motility, progressive motility and concentration. These analyses took approximately 7–8 min during which semen was kept at 37 °C in order to avoid cold shocks. Semen was then pooled and diluted in Tris-egg yolk + glycerol at 30 °C and gradually cooled to 4 °C in 5 h. Afterwards, it was loaded in 0.25 mL straws and frozen in LN_2_. At the time of insemination, straws were thawed in a water bath at 37 °C for 30 sec. The insemination dose was 100 × 10^6^ spz/straw/ewe.

#### 2.4.2. Laparoscopic AI

Ewes were submitted to laparoscopic insemination 58 h after sponge removal and eCG injection. They were previously fasted for at least 12 h. For insemination, ewes were initially submitted to mild sedation with acepromazine maleate (Prequillan, Fatro S.p.A., Ozzano dell’Emilia, Italy; IM, 0.5 mL/50 kg BW) and then placed in a laparoscopic cradle in dorsal recumbency. The abdominal area (from udder to umbilicus) was shaved, cleaned and surgically scrubbed with an antiseptic solution of 10% Povidone Iodide and alcohol. The sites of insertion of trocars and cannulas were identified 5–10 cm cranial to the udder on the left and the right of the middle line and local anaesthesia was provided by injection of 2 mL Lidocaine 2% (Esteve S.p.A., Milan, Italy). Ewes were then placed with the hindquarters elevated of 45°. Trocars and cannulas were inserted through the abdominal wall into the peritoneal cavity that was afterwards inflated with air to enable localization of the reproductive tract with the laparoscope (Richard Wolf, Vernon Hills, IL, USA). The uterus was then placed in a proper position for the insemination with the aid of a palpation probe (Richard Wolf, Vernon Hills, IL, USA) and semen was deposited in both uterine horns with an insemination pipette (Cassou mini-pistolet for ovine-caprine; IMV Technologies, l’Aigle, France) fitted with an Aspic needle (IMV Technologies, l’Aigle, France).

#### 2.4.3. Transcervical AI

Transcervical insemination in ewes submitted to SICF has been previously described by Pau et al. [15]. Briefly, at 58 h after sponge removal and eCG injection, animals were placed in dorsal recumbency and following careful cleaning of the vulvar area a lubricated speculum was gently inserted in the vagina. The fold of the external *os* of the cervix was grasped with forceps and gently retracted caudally toward the vestibulum of the vagina. Insemination was then performed by insertion of the insemination pipette (Cassou mini-pistolet for ovine-caprine; IMV Technologies, l’Aigle, France) through the cervical lumen and semen was deposited directly in the uterine lumen. When the passage of the pipette was difficult, semen was deposited in the cervical lumen as deep as possible. Data on uterine or cervical inseminations were recorded.

### 2.5. Pregnancy and Lambing Rates

Fifteen to twenty-one days after AI, teaser rams were introduced to the two experimental groups to assess the rate of return to oestrus. Pregnancy diagnosis was performed by transrectal ultrasonography (MyLab One, Esaote, Genova, Italy) at 30 days from the AI and pregnancy (PR) [(n. pregnant ewes/n. inseminated ewes) × 100] and lambing (LR) [(number ewes lambing/number inseminated ewes) × 100] were calculated.

### 2.6. Statistical Analysis

The collected data were analysed using Stata 11.2/IC (StataCorp LP, College Station, TX, USA). The Chi-square test for independent samples was used to assess differences between groups in pregnancy and lambing rates. The significance level was defined for a *p* value < 0.05.

## 3. Results

### 3.1. Transcervical Intrauterine Semen Deposition in Ewes Submitted to SICF

At the time of insemination, incisions of cervical folds allowed the passage of the inseminating pipette and consequent uterine deposition of semen in 35/39 ewes (89.7%). In 4/39 ewes (10.3%), semen was deposited in the cervix as deep as possible (Table 1).

### 3.2. Pregnancy and Lambing Rates Following Laparoscopic and Transcervical AI

The overall pregnancy rates, disregarding of the depth of deposition of semen, were similar in ewes inseminated either by laparoscopic AI (70%) or transcervical AI following SICF (71.8%; *p* > 0.05). Lambing rates followed the same trend being 65% and 64.1% respectively in laparoscopic and transcervical groups (*p* > 0.05). When only in utero deposition of semen was considered for ewe transcervically inseminated, pregnancy rates and lambing rates slightly increased being respectively 77.1% and 68.6% and no significant difference was found with the laparoscopic group (*p* > 0.05; Table 1). Only one ewe over the four in which semen was deposited in the cervix was found pregnant and regularly lambed.

## 4. Discussion

The benefits in spreading more efficiently superior genotypes with easy transport and insemination using frozen semen are evident in the bovine species, but very limited in the ovine. Therefore, in this species, the rate of genetic progress could be easily accelerated by optimization of current schemes of artificial insemination. The possibility to deposit FT semen directly into the uterine lumen, avoiding the costs and complications related to laparoscopic AI would provide great advantages. Intrauterine insemination by laparoscopy is still the most widely used for insemination with frozen thawed semen. Pregnancy rates are satisfactory, ranging from 40% [16] to 70%–75% [17,18]. However, there is an increasing demand for a procedure that could be easily applied in field conditions, with no expensive instruments involved and with good pregnancy and lambing rates.

Transcervical insemination after surgical incision of cervical folds might represent a valid alternative to laparoscopic AI. Pau et al. recently described different surgical techniques to remove (totally or partially) the cervical folds or modify their shape (with two or four incisions), with the aim to allow the transcervical passage of an insemination Cassou pipette and the intrauterine deposition of semen [15]. Among the described techniques, the one consisting of four incisions of the cervical folds provided easier access to the uterine lumen, shorter times of insemination and, most importantly, satisfactory pregnancy (63.7%) and lambing (41.4%) rates [15]. In the present study, pregnancy and lambing rates were even higher (71.8% and 64.1%, respectively), suggesting that among other influencing factors, the improvement of the operator skills has beneficial effects on the final results. Based on unpublished observations, surgical manipulation of cervical folds has no long-term consequences on the reproductive career. The ewes selected for the experiment were re-introduced in the original flock and in the following oestrous cycles they showed regular heat behaviour, mated, became pregnant and lambed with no complications. Moreover, their productive performances (milk) were not affected by surgery. These observations strengthen the idea that SICF could be a valuable tool for reproductive purposes in sheep breeding programs.

In our experiment, following surgical incision of the cervical folds, uterine deposition of frozen thawed semen was possible in most of the ewes and the overall pregnancy rate (~72%) was comparable to that obtained with laparoscopic AI (70%). Although the rate increased when considering only intrauterine deposition of semen (~77%), it needs to be pointed out that only one ewe over four in which semen was deposited in the cervical lumen was pregnant and regularly lambed. These findings support the observation that the deeper the semen is deposited in the female genital tract, the higher are the fertility rates [19].

In the past decades, other research groups attempted transcervical AI using FT semen in ovine species with inconsistent results. An encouraging pregnancy rate of 70% was reported in out-of-season synchronised Finncross ewes [20] while Masoudi et al. obtained around 30%–32% pregnancy rates in fat tail Zandi ewes [21]. However, in these studies, the insemination technique used was not described. In the 1990s, Halbert et al. described the “Guelph system for transcervical AI”, consisting of grasping and retracting the cervix with the aid of forceps to ease the passage of an inseminating instrument through the cervical canal of the ewe [14]. Using this technique, pregnancy rates ranged from 20% to 50% when frozen thawed semen was used [4,16,22]. The high variability in the results obtained when transcervical AI is performed using FT semen is probably due to several factors. Differences in breed, age, parity, breeding season, protocols of synchronisation, inseminator skills and insemination technique [1,6,12,23,24] may all affect the degree of transcervical passage of the insemination pipette and consequently the site of deposition of semen. In Sarda ewes, Falaschi et al. reported a field trial using the Guelph system in 296 ewes obtaining lambing rates of around 26% [25]. In the same breed, Cappai et al. compared Guelph transcervical AI with laparoscopic AI and obtained lambing rates of 7% and 62%, respectively [26]. While the results obtained with the laparoscopic technique were similar to those reported in the literature [18,27,28] and to what was observed in the present trial (~65%), the authors explained the poor results of transcervical AI with the possible puncturing and bruising of the cervical canal during the insemination, which may have compromised the success of the technique [26].

Although SICF involves surgery, it is a permanent, once in a lifetime procedure that allows uterine catheterisation for all the reproductive career of the animal (unpublished data). This technique would provide great benefits when applied for the insemination of small nuclei of selected dams with FT semen of superior sires. Moreover, SICF is intended to be used in future to allow MOET (multiple ovulation and embryo transfer) via cervical passage of catheters (for insemination, flushing and transfer), rather than via laparotomy, with gains in terms of animal welfare and easiness in spreading superior genotypes in the sheep breeding system.

In practice, transcervical AI in SICF ewes may represent a faster and more economical technique compared to laparoscopic AI. The time employed to pass the cervix in SICF ewes is around 21 s [15] which is a considerably short time compared to that of laparoscopic AI of around 3 to 4 min/ewe (based on our experience and including trocar insertion). Moreover, the costs of the two procedures may significantly differ. As recently reviewed by Sathe (2018), equipment for laparoscopic insemination may cost around 10,500 to 15,000 USD [29] and it requires trained and experienced veterinarians. Transcervical insemination after SICF could be easily performed by the farmer themselves, and the majority of the costs could be attributed to vet fees for the surgery, which itself does not require any specific equipment apart from a basic surgical kit and proper sedatives and anaesthetics of common use in veterinary practice.

## 5. Conclusions

Surgical incision of cervical folds offers great advantages in the use of AI protocols with frozen thawed semen, obtaining pregnancy rates that are comparable to those achieved by laparoscopic AI. This technique facilitates the transcervical passage of an insemination pipette and intrauterine semen deposition. Although it is a surgical procedure, it is permanent and might allow transcervical insemination during the reproductive life of the animal. Further investigations will focus on the use of SICF for MOET protocols.

## Figures and Tables

**Table 1:** Pregnancy (PR) and lambing rates (LR) in ewes submitted either to laparoscopic AI or transcervical AI after SICF (surgical incision of the cervical folds).

Insemination	Cervical Deposition of Semen			In Utero Deposition of Semen			Overall		
Method	n	PR	LR	n	PR	LR	n	PR	LR
**Laparoscopic AI**	-			40	70%	65%	40	70%	65%
**Transcervical AI**	4	25%	25%	35	77.10%	68.60%	39	71.80%	64.10%
**after SICF**									
